# Overexpression of miR-506-3p Aggravates DBP-Induced Testicular Oxidative Stress in Rats by Downregulating ANXA5 via Nrf2/HO-1 Signaling Pathway

**DOI:** 10.1155/2020/4640605

**Published:** 2020-11-28

**Authors:** Min Tang, Lei Zhang, Zheng Zhu, Ran Li, Shangqian Wang, Wei Wang, Zhiqiang Qin, Wei Zhang

**Affiliations:** ^1^Department of Urology, The First Affiliated Hospital of Nanjing Medical University, Nanjing 210009, China; ^2^First Clinical Medical College of Nanjing Medical University, Nanjing 210009, China; ^3^Department of Urology and Transplantation, Nanjing First Hospital, Nanjing Medical University, Nanjing 210006, China

## Abstract

**Background:**

Di-N-butylphthalate (DBP) is a kind of unique endocrine toxicity linked to hormonal disruptions that affects the male reproductive system and has given rise to more and more attention. However, the mechanism of DBP-induced testicular injury remains unclear. Here, the objective of this study was to investigate the potential molecular mechanism of miR-506-3p in DBP-induced rat testicular oxidative stress injury via ANXA5 (Annexin A5)/Nrf2/HO-1 signaling pathway.

**Methods:**

*In vivo*, a total of 40 adolescent male rats were treated from 2 weeks with 800 mg/kg/day of DBP in 1 mL/kg corn oil administered daily by oral gavage. Among them, some rats were also injected subcutaneously with 2 nmol agomir-506-3p and/or 10 nmol recombinant rat ANXA5. The pathomorphological changes of testicular tissue were assessed by histological examination, and the antioxidant factors were evaluated. Subsequently, ANXA5, Nrf2, and its dependent antioxidant enzymes, such as HO-1, NQO1, and GST, were detected by Western blotting or immunohistochemical staining. *In vitro*, TM3 cells (Leydig cells) were used to detect the cell activity by CCK-8 and the transfection in the DBP-treated group.

**Results:**

Differentially expressed miRNAs between the DBP-treated and normal rats were analyzed, and qRT-PCR showed miR-506-3p was highly expressed in testicular tissues of the DBP-treated rats. DBP-treated rats presented severe inflammatory infiltration, increased abnormal germ cells, and missed cell layers frequently existed in seminiferous tubules, resulted in oxidative stress and decreased testicular function. Meanwhile, upregulation of miR-506-3p aggravated the above changes. In addition, miR-506-3p directly bound to ANXA5, and overexpression of miR-506-3p could reduce the ANXA5 expression and also decrease the protein levels of Nrf2/HO-1 signaling pathway. Additionally, we found that recombinant rat ANXA5 reversed the DBP-treated testicular oxidative stress promoting injury of miR-506-3p in rats. *In vivo* results were reproduced in *in vitro* experiments.

**Conclusions:**

This study provided evidence that miR-506-3p could aggravate the DBP-treated testicular oxidative stress injury *in vivo* and *in vitro* by inhibiting ANXA5 expression and downregulating Nrf2/HO-1 signaling pathway, which might provide novel understanding in DBP-induced testicular injury therapy.

## 1. Introduction

As one kind of phthalic acid esters (PAEs), Di-N-butylphthalate (DBP) is worldwide used in industrial products [1, 2]. In 2015, the annual production of phthalate is in the range of 5-8 million tons all over the world [3]. Nevertheless, DBP can cause the pollution of surrounding soil and atmosphere through continuous leaching with its noncovalently bounding to the plastic matrix, which contributes to human intake [4, 5]. Long-term exposure to DBP can induce serious injuries to various organs, especially to reproductive system [6, 7]. A critical association between maternal DBP exposure and androgen disruption has been well documented in male infants [7]. Also, testicular cell dysplasia, abnormal cholesterol transport and steroidogenesis, and low testosterone production accompanied by demasculinization have been reported in rodents prenatally exposed to DBP [8–12]. Additionally, utero exposure during embryonic will destruct the formation of urethra together with the differentiation and development of reproductive nodule, leading to a series of congenital malformation including hypospadias, cryptorchidism, and so on [13, 14]. Hence, with limited knowledge of the physiological toxicity and ecotoxicity in DBP, it is worthy of in-depth study on the damage to human reproductive system and the specific mechanism of such injuries.

Previous studies have demonstrated DBP could induce oxidative stress *in vivo* [15, 16]. Transcriptional factor nuclear factor erythroid-related factor 2, also abbreviated as Nrf2, is a main regulator in antioxidant course [17, 18]. Through translocating to the nucleus and then combining to antioxidant response element (ARE), Nrf2 triggers the transcription of downstream endogenous antioxidative genes including but not limited to heme oxygenase 1 (HO-1), nicotinamide adenine dinucleotide phosphate quinone oxidoreductase 1 (NQO1), and antagonizing injuries caused by oxidative stress [19, 20]. What is more, we have verified this conclusion *in vitro* with DBP administration in mouse testicular Leydig cells and found that oxidative stress played a significant role in the damage originating from DBP and provided promising prevention measures to its toxicity by decreasing the protein expression levels of Nrf2/HO-1 pathway [21, 22].

microRNA (miRNA), as a branch of small noncoding RNAs (ncRNAs), participates in the regulation of posttranscriptional gene expression by binding with target mRNA [23, 24]. Increasing studies have indicated that miRNAs played vital roles in various pathophysiological processes [25, 26]. Meanwhile, previous studies have found dissimilar expressed miRNAs between these rats exposed to DBP and the normal rats [27, 28]. However, the mechanism how miRNA functions, particularly in the progression of DBP-induced testicular injury, has not yet been clarified. Herein, we pioneered the researches on the expression profile, biological regulation, and detailed molecular mechanisms of a new identified miRNA (miR-506-3p) in DBP-induced testicular injury. Abundant real-time PCR (qRT-PCR) assays have testified significantly upregulated the expression levels of miR-506-3p in DBP-mediated testicular injury. Furthermore, our findings indicated that miR-506-3p downregulated the expression of ANXA5 via Nrf2/HO-1 signaling pathway in rats and provided a novel potential therapeutic target for DBP-induced testicular injury.

## 2. Materials and Methods

### 2.1. Animals and Experimental Protocol

All procedures conducted in experimental animals, and the protocols were approved by the Committee on the Ethics of Animal Research in Animal Care Facility of Nanjing Medical University (Nanjing, Jiangsu, China). The study was carried out in strict accordance with the recommendations in the Guide for the Care and Use of Laboratory Animals of the National Institutes of Health. 40 adolescent male Sprague-Dawley (SD) rats (180-220 g) at the age of averaging 6 weeks old were purchased from the Animal Experiment Center of Nanjing Medical University (Nanjing, Jiangsu, China). These animals were maintained at room temperature (23 ± 2°C), relative 50 ± 10% humidity in an automatically controlled 12 hours light/dark cycle environment. Besides, these rats were supplied with abundant pellet chow and water during this experiment.

### 2.2. Animal Treatment

The adolescent male SD rats were housed at a constant temperature, with a 12 : 12 h light-dark cycle and free access to water and rodent food. All protocols were approved by the Committee on the Ethics of Animal Experiments of Nanjing Medical University (Nanjing, China). DBP (Sigma-Aldrich; St Louis, Missouri, USA) was suspended in corn oil (vehicle control, 99.5% pure, Solvent Factory, Shanghai, China) for gavage. DBP gavage dose and time-window selection were administrated according to our previous study [26]. The male SD rats were randomly assigned to 5 groups (*n* = 8) and administered corn oil (vehicle control) or DBP from 2 weeks with 800 mg/kg/day of DBP in 1 mL/kg corn oil administered daily by oral gavage. After 2 weeks of treatment, all testes of rats were carefully removed, and one of testes was stored in 4% formaldehyde, and the other one was kept at -80°C for subsequent biochemical measures. In addition, the sperms were incubated in normal saline with 5% BSA for further analysis.

### 2.3. Cell Culture and Transfection

Mouse Leydig TM3 cell line was obtained from the cell bank of Chinese Academy of Science (Shanghai, China). TM3 cells were cultured in Dulbecco's modified Eagle's and Ham's F12 (DME/F12 1 : 1, *vol*/*vol*) medium (PAA, Germany) and 10% fetal bovine serum (PAA). The 0, 5, 10, and 50 mg/mL DBP were prepared and dissolved in the dimethyl sulfoxide (DMSO, Sigma, USA). Then, these cells were, respectively, treated with 1 *μ*l DBP diluent per 1 mL culture medium, and the total volume of the DBP diluent was kept consistent in each well. In addition, transfection was performed to upregulate the expression levels of miR-506-3p. Briefly, dissolving miR-506-3p mimic or negative control mimic in Opti-MEM separately. The solutions were equilibrated for 5 mins at room temperature. Then, according to the manufacturer's protocol, combining each solution with Lipofectamine 3000 transfection reagent and mixing the solution gently allowed to form inhibitor liposomes for 20 mins.

### 2.4. Cell Viability Assay

Cells (10^4^ cells/well) were plated onto 96-well round-bottom plates and treated with DBP at various concentrations. After 24 h, Cell Counting Kit-8 (CCK-8) reagents (Dojindo Laboratories, Kumamoto, Japan) were added to each well and incubated for 2 h. The absorbance at 450 nm was measured using a microplate reader (Tecan, Männedorf, Switzerland). Finally, cell viability (%) was calculated by a specific formula: (the mean OD value of treatment group − mean OD value of blank)/(the mean OD value of control group − mean OD value of blank)∗100%. The cell viability of control group was adjusted to 100%.

### 2.5. Antioxidant Capacity Assays

The levels of malondialdehyde (MDA), catalase (CAT), total antioxidant capacity (T-AOC), superoxide dismutase (SOD), glutathione (GSH), and reduced GSH were determined by using each assay kit (Jiancheng Bioengineering Institute, Nanjing, China), according to the manufacturer's instructions.

### 2.6. Reactive Oxygen Species (ROS) Level Detection

ROS level assay was conducted using in situ dihydroethidium (DHE, Sigma-Aldrich, USA) fluorescence. After being fixed by methanal, cells were incubated with DHE in a light-protected, humidified chamber at room temperature, and then, cell nucleus were stained with DAPI. ROS was observed under a fluorescent microscope (Eclipse Ti-SR, Nikon Co., Japan), and the density of images was detected in arbitrary units per millimetre square field by a fluorescence spectrophotometer.

### 2.7. miRNA Microarray Assay

According to the manufacturer's instructions, total RNA samples from the DBP-treated group and the normal group of rats (*n* = 3) were isolated using TRIzol reagent (Invitrogen, USA) and purified using RNeasy mini kit (Qiagen, Germany). The hierarchical clustering was performed to show the distinguishable miRNA expression profiling among samples.

### 2.8. Prediction of Target miRNAs and Dual-Luciferase Reporter Assay

The target miRNAs of ANXA5 validated above were screened by the “miRDB,” “TargetScan,” and “StarBase” databases, and each target miRNA must be searched in at least two databases. Establishing the plasmids containing the wild-type miR-506-3p-ANXA5 (wt-Luc-ANXA5) response element and the corresponding mutant (mut-Luc-ANXA5), the blank plasmids were purchased from RiboBio. Co., Ltd. (Guangzhou, China). Cotransfecte Plasmid DNA (wt-Luc-ANXA5, mut-Luc-ANXA5) and miR-506-3p mimic or NC mimic into TM3. Finally, the luciferase activity with a Double-Luciferase Reporter Assay Kit which purchased from Promega Biotech Co., Ltd. (Beijing, China) and using the Dual-Light Chemiluminescent Reporter Gene Assay System (Berthold, Germany) was assessed to firefly luciferase activity.

### 2.9. Histological Examination

Testicular tissue samples were fixed in 4% paraformaldehyde for 24 hours, dehydrated, embedded in paraffin, sectioned at 5 *μ*m, and stained with hematoxylin and eosin. Next, the sections were evaluated under a standard light microscopy (Olympus BX-51, Tokyo, Japan) for the change of testis structural by two blinded investigators. The mean seminiferous tubular diameter (MSTD) of the seminiferous tubules was measured in the same histological section on 5 different focuses with a microscope-adaptable micrometer.

### 2.10. Immunohistochemical Staining

Testicular tissue sections were fixed with 4% paraformaldehyde for 30 min at room temperature. After slides were microwaved for 20 min and allowed to cool for 1 h at room temperature, endogenous peroxidase activity was blocked in all sections by incubating the sections in 3% H_2_O_2_ for 15 minutes. Then, sections were incubated with anti-Nrf2 antibody (1 : 500) or anti-ANXA5 antibody (1 : 500) for 1 h, washed extensively, and stained for 1 h with either Alexa Fluor 594- or Texas Red-conjugated goat anti-rabbit IgG (1 : 1000). The immunohistochemical staining was analyzed using a Zeiss LSM 510 META confocal laser scanning microscope.

### 2.11. Quantitative Real-Time PCR (qRT-PCR)

Total RNA was extracted from cultured TM3 cells by TRIzol reagent (Invitrogen, Carlsbad, CA, USA), and then, cDNA was reverse transcribed with Primescript RT Reagent (Takara, Otsu, Japan) according to the manufacturer's instructions. Then, qRT-PCR was performed by using StepOne Plus Real-time PCR system (Applied Biosystems, Foster City, CA, USA) with SYBR® Premix Ex Taq™ Reagent (Takara). Following distinct primers designed and synthesized by Sangon Biotech (China) were used in this study: miR-506-3p: Forward: 5′-GCCACCACCATCAGCCATAC-3′, Reverse: 5′-GCACATTACTCTACTCAGAAGGG-3′; U6: Forward: 5′-TCCGATCGTGAAGCGTTC-3′, Reverse: 5′-GTGCAGGGTCCGAGGT-3′; ANXA5: Forward: 5′-AGCGGGCTGATGCAGAAAC-3′, Reverse: 5′-ACTTCGGG ATGTCAACAGAGT-3′; and GAPDH: Forward: 5′-AAGGTGAAGGTCGGAGTCAA-3′, Reverse: 5′-AATGAAGGGGTCATTGATGG-3′.

The ABI Step One Software version 2.1 was utilized to conduct data analysis, and the relative mRNA level was calculated using the 2^-*ΔΔ*Ct^ method.

### 2.12. Western Blot Analysis

Total protein concentration was calculated by the BCA Protein Assay kit (Pierce, Rockford, IL, USA), according to the manufacturer's instructions. The cells and tissues were lysed in cell lysis buffer for 20 min vibration on the ice and centrifuged at 12.000 × g at 4°C for 15 min. Proteins were separated upon 10% SDS-PAGE and transferred onto 0.45 mm PVDF membrane (Bio-Rad, California, Hercules, USA). The membranes were incubated with 5% nonfat milk powder which dissolved in TBST (20 mM Tris-HCL, pH 7.5, 150 mM NaCl, 0.1% Tween 20) for 2 hours. The following protein antibody Nrf2 (68.0 kDa), Histone H3 (15.0 kDa), and GAPDH (40.2 kDa) (Cell Signaling Technology, CST); HO-1 (34.6 kDa), NQO-1 (30.0 kDa), and GST (24.0 kDa) (Abcam); and ANXA5 (36.0 kDa) (Santa Cruz, CA) were used to bind with corresponding proteins and incubated overnight at 4°C. All these protein antibodies were diluted by the Antibody Dilution Buffer (Beyotime, China) with a ratio of 1 : 1000-2000. HRP-conjugated secondary antibody (1 : 4000, Cell Signaling Technology, USA) was used to bind with primary antibodies for about 1.5 hours. Finally, immunoreactive bands were visualized with electrochemiluminescence reagent (Amersham, Uppsala, Sweden). Densitometry and quantitative analysis were analyzed using the ImageJ software (Bethesda, USA) and regulated by Histone H3 and GAPDH.

### 2.13. Statistical Analyses

Statistical analyses were performed using the SPSS 22.0 (Armonk, New York, USA). The results are expressed as the mean ± standard deviation (SD) for each group. Analysis of variance (ANOVA) was used for multiple comparisons unpaired with Student's *t*-test, to evaluate the significance of differences. *P* < 0.05 was considered significant.

## 3. Results

### 3.1. miR-506-3p Was Highly Expressed in DBP-Induced Testicular Injury in Rats

**Figure 1:**
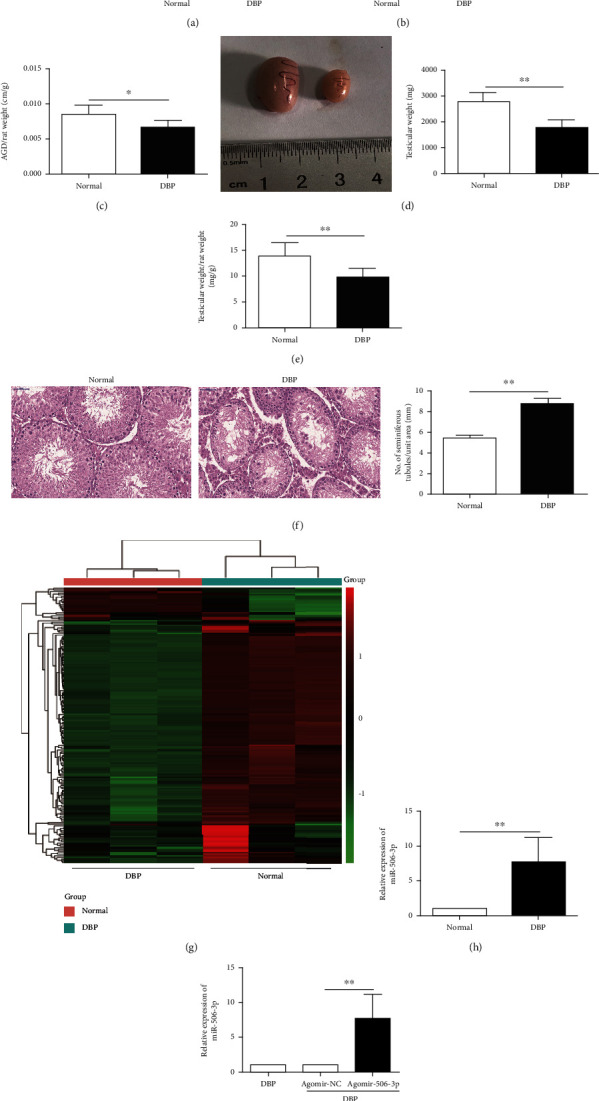
miR-506-3p was highly expressed in DBP-induced oxidative stress injury in rats: (a) body weight of these rats treated with the DBP group and the normal group; (b) the AGD in these rats treated with the DBP group and the normal group; (c) the ratio of AGD/rat weight in these rats treated with the DBP group and the normal group; (d) images of gross morphology of the testes and testicular weight in these rats treated with the DBP group and the normal group; (e) the ratio of testicular weight/rat weight in these two groups; (f) H&E staining of testis tissues in DBP-treated rats and normal rats (×400); (g) hierarchical clustering analysis of the differentially expressed miRNAs of testis samples in these rats treated with the DBP group and the normal group; (h) miR-506-3p expression level in these rats treated with the DBP group and the normal group using RT-qPCR; (i) miR-506-3p expression level in testis tissues of DBP-treated rats following miR-506-3p agomir and NC agomir injection detected using RT-qPCR. The data are showed as mean ± SD. ^∗^Significantly different from the normal group. ^∗^*P* < 0.05, ^∗∗^*P* < 0.01; ns indicated no significance.

As revealed in Table 1, DBP could reduce the sperm count and sperm viability of rats and increase the malformation rate. Besides, the level of serum testosterone (T) decreased whereas follicle stimulating hormone (FSH) and luteinizing hormone (LH) increased in the DBP-treated group, compared with the normal group. The rat weight of the rats did not differ between the normal group and the DBP group (Figure 1(a)). The significantly decreased anogenital distance (AGD) and the ratio of AGD/rat weight were observed in the DBP-treated group, compared with normal group (Figures 1(b) and 1(c)). In addition, the testicular weight and the ratio of testicular weight/rat weight were also lower than the DBP-treated group, and the results were statistically significant (Figures 1(d) and 1(e)). H&E staining showed DBP significantly aggravated testicular seminiferous epithelium injury, increased abnormal germ cells, and missed cell layers frequently existed in seminiferous tubules (Figure 1(f)).

A total of these differentially expressed miRNAs with at least 2-fold changes and *P* values less than 0.05 were identified in testis tissues of rats caused by DBP-induced testicular injury using microRNA microarray analysis, compared with the normal group. As shown in Figure 1(g), the heat map indicated the results of a two-way hierarchical clustering of the samples and these differentially expressed miRNAs, which displayed the relative expression levels identified by microarray assay. Amongst them, miR-506-3p was found the obvious differences between the normal group and the DBP group, and DBP could significantly increase the expression level of miR-506-3p in rats (Figure 1(h)). Thus, it could be inferred that upregulation of miR-506-3p might participate in DBP-induced testicular injury. Thereby, the DBP-treated rats were injected with miR-506-3p agomir to further identify the roles of miR-506-3p in DBP-treated rats. The qRT-PCR result presented that the DBP-treated rats with agomir-506-3p could significantly increase the expression level of miR-506-3p, compared to these DBP-treated rats with agomir-NC (Figure 1(i)).

### 3.2. Upregulation of miR-506-3p Aggravated Testicular Oxidative Stress Injury in DBP-Treated Rats

**Figure 2:**
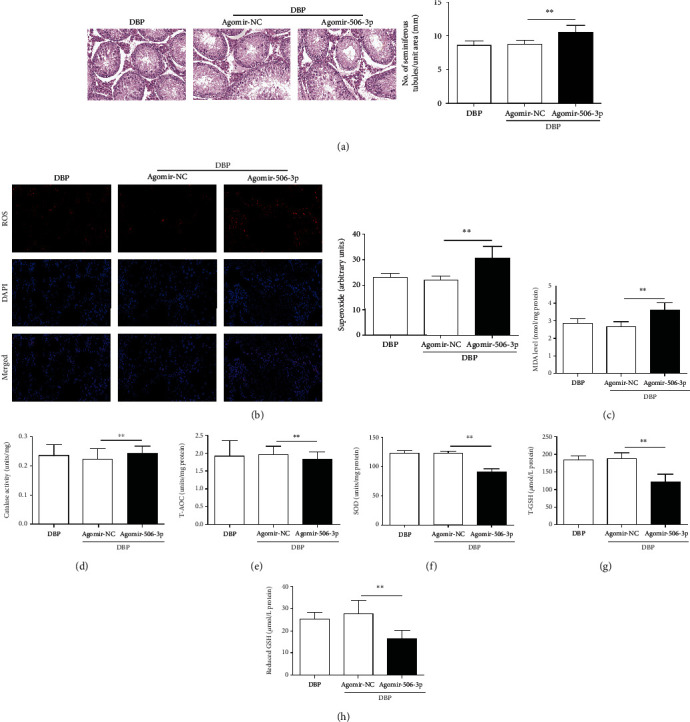
Upregulation of miR-506-3p aggravated testicular oxidative stress injury in DBP-treated rats: (a) H&E staining of testis tissues in DBP-treated rats following miR-506-3p agomir and NC agomir injection; (b) DHE staining of testis tissues of DBP-treated rats following miR-506-3p agomir and NC agomir injection, ROS exhibit red fluorescence under fluorescent microscope; (c–h) content of MDA, CAT, T-AOC, SOD, T-GSH, and reduced GSH of testis tissues in each group. The data are showed as mean ± SD. ^∗^Significantly different from the DBP + Agomir-NC group. ^∗∗^*P* < 0.01.

Following agomir-506-3p injection, H&E staining was performed to measure the morphological changes in testicular tissues of rats. As mentioned above, the testicular tissues of DBP-treated rats were disorganized with the cell layers of seminiferous tubules irregularly arranged or even missing, while upregulation of miR-506-3p increased the swelling and inflammatory cell infiltration in DBP-treated rats (Figure 2(a)). Moreover, ROS staining results identified that upregulation of miR-506-3p enhanced the intensity of the DHE (dihydroethidium) staining of testis in DBP-treated rats (Figure 2(b)). Thereafter, oxidative stress was evaluated in rat testicular tissues through the levels of MDA, CAT, T-AOC, SOD, GSH, and reduced GSH. As shown in Figures 2(c)–2(h), the MDA content in the DBP + Agomir-506-3p group was significantly higher compared to the DBP + Agomir-NC group (*P* < 0.05). In addition, miR-506-3p agomir could decrease the level of antioxidant enzymes SOD and CAT, and the content of antioxidants T-AOC and GSH, GSH/GSSG to aggravate testicular oxidative stress compared to the DBP + Agomir-NC group. Thus, the above results indicated that miR-506-3p aggravated DBP-induced testicular oxidative damage *in vivo*.

### 3.3. Upregulation of miR-506-3p Decreased the Expression Levels of Nrf2 and Its Downstream Target Genes in DBP-Treated Rats

**Figure 3:**
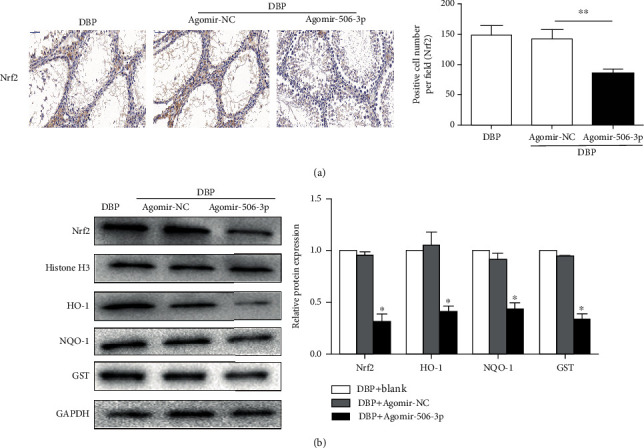
Upregulation of miR-506-3p decreased the expression levels of Nrf2 and its downstream target genes in DBP-treated rats. (a) Immunohistochemical staining showed Nrf2 expression level of testis tissues in DBP-treated rats following miR-506-3p agomir and NC agomir injection. (b) Western blotting showed the expression levels of Nrf2 and its downstream target genes, HO-1, NQO1, and GST of testis tissues in DBP-treated rats following miR-506-3p agomir and NC agomir injection. The data are showed as mean ± SD. ^∗^Significantly different from the DBP + Agomir-NC group. ^∗∗^*P* < 0.01.

The expression level of Nrf2 observed in the testes as assessed from immunohistochemical analysis was as follows. Compared to the DBP group and the DBP + Agomir-NC group, the expression level of Nrf2 was decreased in the DBP + Agomir-506-3p group (*P* < 0.05, Figure 3(a)). As shown in Figure 3(b), the expression levels of Nrf2, HO-1, NQO1, and GST proteins were analyzed by Western blotting. The expression levels of Nrf2 and its downstream target genes, HO-1, NQO1, and GST, in the DBP + Agomir-506-3p group were significantly decreased compared with the DBP group and the DBP + Agomir-NC group (*P* < 0.05). Therefore, upregulation of miR-506-3p led to decrease the expression levels of Nrf2 and its downstream target genes in DBP-treated rats.

### 3.4. miR-506-3p Was Bound to ANXA5

**Figure 4:**
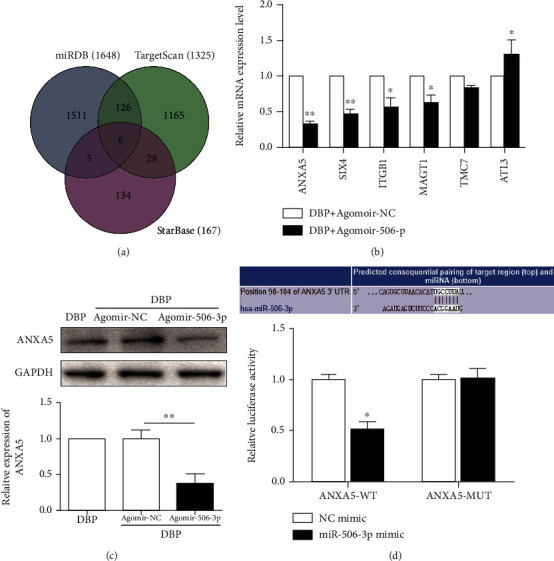
miR-506-3p was bound to ANXA5: (a) a schematic diagram used to search the target mRNAs of miR-506-3p in three databases; (b) validation of the six differently expressed mRNAs in DBP-treated rats following miR-506-3p agomir and NC agomir injection based on qRT-PCR assay; (c) Western blotting showed the expression levels of ANXA5 of testis tissues in DBP-treated rats following miR-506-3p agomir and NC agomir injection; (d) the relative luciferase expression with ANXA5 3′-UTR after cotransfection with miR-506-3p mimic or NC mimic in TM3. The data are showed as mean ± SD. ^∗^Significantly different from the DBP + Agomir-NC group. ^∗^*P* < 0.05, ^∗∗^*P* < 0.01.

As shown in Figure 4(a), the interaction networks between the miR-506-3p and its downstream mRNAs were predicted by Mirbase (http://www.mirbase.org/), Mirdb (http://www.mirdb.org/), and MiRanda (http://www.microrna.org/microrna/home.do). Then, six mRNAs were further validated by qRT-PCR assay. As shown in Figure 4(b), miR-506-3p significantly decreased the expression levels of ANXA5, SIX4, ITGB1, and MAGT1 and obviously increased the level of ATL3 in DBP-treated rats, compared with the Agomir-NC group. In addition, the result of Western blotting also showed that compared with the Agomir-NC group, miR-506-3p could significantly decreased the ANXA5 expression level in DBP-treated rats (Figure 4(c)). The RNA sequence alignment showed that the 3′-UTR of ANXA5 mRNA contained a complementary site for the seed region of miR-506-3p. As presented in Figure 4(d), the dual luciferase reporter plasmid was obtained to perform the dual-luciferase reporter assay. In the groups of ANXA5-Wt, the luciferase activities were significantly repressed by miR-506-3p mimic compared with the NC mimic group. However, these effects were not observed with the mutated ANXA5 group, suggesting that ANXA5 was the target gene of miR-506-3p.

### 3.5. Recombinant Rat ANXA5 Reversed the Testicular Oxidative Stress Promoting Injury of miR-506-3p in DBP-Treated Rats

**Figure 5:**
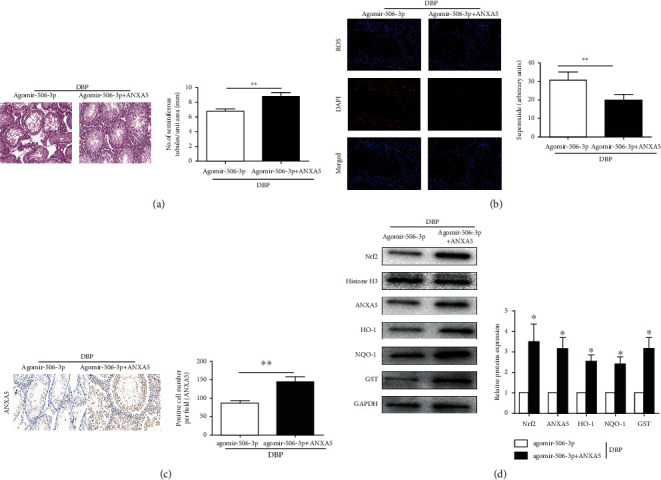
Recombinant rat ANXA5 reversed the testicular oxidative stress promoting injury of miR-506-3p in DBP-treated rats: (a) H&E staining of testis tissues in DBP-treated rats following Agomir-506-3p and cotransfection of recombinant rat ANXA5 and Agomir-506-3p; (b) DHE staining of testis tissues in DBP-treated rats following Agomir-506-3p and cotransfection of recombinant rat ANXA5 and Agomir-506-3p, ROS exhibit red fluorescence under fluorescent microscope; (c) immunohistochemical staining showed ANXA5 expression level of testis tissues in DBP-treated rats following Agomir-506-3p and cotransfection of recombinant rat ANXA5 and Agomir-506-3p; (d) Western blotting showed the expression levels of Nrf2, ANXA5, HO-1, NQO1, and GST proteins of testis tissues in DBP-treated rats following Agomir-506-3p and cotransfection of recombinant rat ANXA5 and Agomir-506-3p. The data are showed as mean ± SD. ^∗^Significantly different from the DBP + Agomir-506-3p group. ^∗^*P* < 0.05, ^∗∗^*P* < 0.01.

To further confirm if ANXA5 activation was responsible for testicular oxidative stress promoting injury of miR-506-3p, the DBP-treated rats were further cotransfected with miR-506-3p agomir and recombinant rat ANXA5. The results suggested that compared to miR-506-3p transfection, cotransfection of recombinant rat ANXA5 and Agomir-506-3p led to preserve the tubular structure and arrangement of the spermatogonial cells (Figure 5(a)). Meanwhile, the intracellular ROS in DBP-treated rats with transfected with cotransfection of recombinant rat ANXA5 and Agomir-506-3p was significantly lower than the DBP + Agomir-506-3p group, which indicated that ANXA5 could reversed the increased intracellular ROS caused by miR-506-3p in DBP-treated rats (Figure 5(b)). In addition, immunohistochemical analysis showed that compared to the DBP + Agomir-506-3p group, the expression level of ANXA5 was increased in DBP-treated rats with cotransfection of recombinant rat ANXA5 and Agomir-506-3p (Figure 5(c)). As shown in Figure 5(d), the expression levels of Nrf2, ANXA5, HO-1, NQO1, and GST proteins were analyzed by Western blotting. The expression levels of ANXA5 and Nrf2 as well as its downstream target genes HO-1, NQO1, and GST in the cotransfection of recombinant rat ANXA5 and Agomir-506-3p were significantly increased compared with the DBP + Agomir-506-3p group (*P* < 0.05). Therefore, the above results suggested that recombinant rat ANXA5 could reverse the decreased expression levels of Nrf2 and its downstream target genes caused by upregulation of miR-506-3p in DBP-treated rats.

### 3.6. mir-506-3p Aggravated the Oxidative Stress of DBP-Treated TM3 Cells by Downregulating ANXA5 *In Vitro*

**Figure 6:**
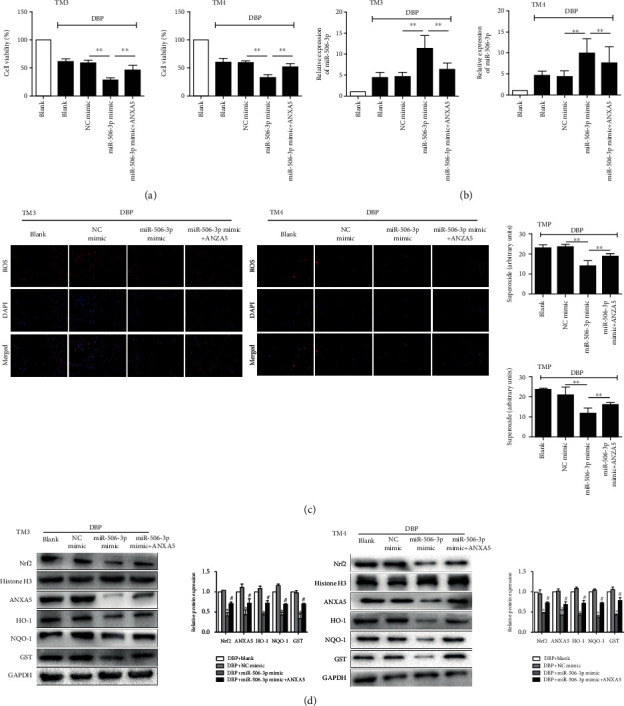
miR-506-3p aggravated the oxidative stress of DBP-treated TM3 cells by downregulating ANXA5 *in vitro*: (a) cell viability after 24 h of 10 mg/L DBP administration after cotransfection of recombinant rat ANXA5 and miR-506-3p mimic in TM3 and TM4 cells; (b) qRT-PCR showed the expression level of miR-506-3p of testis tissues after 24 h of 10 mg/L DBP administration after cotransfection of recombinant rat ANXA5 and miR-506-3p mimic in TM3 and TM4 cells; (c) DHE staining of testis tissues after 24 h of 10 mg/L DBP administration after cotransfection of recombinant rat ANXA5 and miR-506-3p mimic in TM3 and TM4 cells; (d) Western blotting showed the expression levels of Nrf2, ANXA5, HO-1, NQO1, and GST proteins of testis tissues after 24 h of 10 mg/L DBP administration after cotransfection of recombinant rat ANXA5 and miR-506-3p mimic in TM3 and TM4 cells. The data are showed as mean ± SD. ^∗^Significantly different from NC mimic. ^#^Significantly different from miR-506-3p mimic. ^∗^^#^*P* < 0.05, ^∗∗^*P* < 0.01.

The viability of TM3 cells was examined by CCK-8 after treated with different concentration of DBP (0, 5, 10, 50 mg/L) after 24 hours. The results found that the difference was significant (*P* < 0.05) when the TM3 and TM4 cells were treated with 10 and 50 mg/L DBP, while no obvious change was found in the DBP 5 mg/L group. Eventually, 10 mg/L DBP was chosen in this study. To investigate the interaction of miR-506-3p and ANXA5 in DBP-induced oxidative damage, recombinant rat ANXA5 was administrated, and miR-506-3p mimic was transfected to TM3 and TM4 cells in DBP model. Overexpression of miR-506-3p could reduce the viability of TM3 and TM4 cells; however, recombinant rat ANXA5 could reverse the decreased of the viability of TM3 and TM4 cells caused by overexpression of miR-506-3p *in vitro*, thus to increase the viability in DBP-treated TM3 and TM4 cells (Figure 6(a)). Next then, we tested the expression levels of miR-506-3p, and the results found recombinant rat ANXA5 could decrease the expression level of miR-506-3p (Figure 6(b)). Subsequently, we could show clear in Figure 6(c) that overexpression of miR-506-3p could increase the intracellular ROS in TM3 and TM4 cells with DBP treatment (10 mg/L); but, the cotransfection of recombinant rat ANXA5 and miR-506-3p mimic significantly reversed the increased intracellular ROS caused by overexpression of miR-506-3p in DBP-treated TM3 and TM4 cells. As shown in Figure 6(d), the levels of ANXA5 and Nrf2 as well as its downstream target genes HO-1, NQO1, and GST were detected by Western blotting in DBP TM3 and TM4 cells model. The results found that overexpression of miR-506-3p notably significantly decreased ANXA5 and Nrf2 as well as its downstream target genes. However, recombinant rat ANXA5 could reverse the promoting effect on Nrf2/HO-1 pathway caused by overexpression of miR-506-3p *in vitro*. All results suggested that miR-506-3p aggravated the oxidative stress of DBP-treated rats by downregulating ANXA5 via Nrf2/HO-1 signaling pathway.

## 4. Discussion

DBP exposure, generally through the route of food and water, could cause damage to various human body systems, especially to children [4, 5]. With the characteristic estrogenic activity, the toxicity of DBP is manifested as low level androgen synthesis and male reproduction dysfunction mentioned before [3, 7, 8]. Apart from the disturbance of androgen metabolism, DBP also generates directly toxicity to cell or tissue through multiple damage pathway, which has been proved that DBP could induce remarkable oxidative stress damage to animal testis [15, 16]. When testicular cells were attacked by DBP-induced oxidative stress, Nrf2 antioxidation pathway which was regarded as the primary cellular defense against oxidative stress was compensatorily activated to resist such injury [17–20].

Through years of studies on miRNAs, the participation of its aberrant expression has been reported in various pathophysiological processes [25, 26]. Accounting for the significant associations between the aberrant expression of miRNAs and the developmental malformations including which caused by DBP-induced testicular oxidative stress, increasingly more researchers have focused on the utilization of miRNA as a novel marker for the prognostic and treatment of DBP-induced testicular injury [27–29]. In the current study, microRNA microarray was used to screen out the differentially expressed microRNAs in testis tissues of rats caused by DBP-induced testicular injury, and miR-506-3p was aberrantly upregulated in DBP-induced testicular oxidative stress, as well as DBP-treated TM3 cell model. miR-506-3p has also been confirmed indispensable in inflammatory diseases. However, no research ever reporting the effects of miR-506-3p on DBP-induced testicular oxidative stress injury was found, and thus, miR-506-3p might be considered as one potential therapeutic target to aggravate DBP-induced testicular injury. Besides, upregulation of miR-506-3p enhanced the intensity of the ROS staining and the MDA content of testis tissues in DBP-treated rats. In addition, miR-506-3p agomir could decrease the level of antioxidant enzymes SOD and CAT, and the content of antioxidants T-AOC and GSH, GSH/GSSG to aggravate testicular oxidative stress injury compared to the DBP + Agomir-NC group. Hence, the above results indicated that miR-506-3p aggravated DBP-induced testicular oxidative damage *in vivo*.

Based on bioinformatics analysis and double-luciferase reporter assay, the results showed that miR-506-3p could directly target ANXA5. Thus, miR-506-3p might be bound to ANXA5 through adjusting Nrf2/HO-1 pathway to aggravate DBP-induced testicular oxidative damage injury. Annexin A5 (ANXA5), a 35 kDa human protein, functions as an endogenous regulator in various pathophysiological processes through binding to phosphatidylserine (PS) in a Ca^2+^-dependent manner [30, 31]. A few researches have indicated that ANXA5 was related to inflammatory response and cell apoptosis [32, 33]. Yao et al. found that ANXA5 was highly expressed in rat Leydig cells and could interfere in the regulation of testosterone synthesis and secretion [34]. In addition, Ewing et al. found that ANXA5 might exert inflammation and antioxidant function in some cases [35]. Therefore, this study is aimed at exploring the definite role of ANXA5 playing in DBP-induced oxidative stress injury in rat testicular cells. In view of the similar variation trend of miR-506-3p and ANXA5 after DBP administration, we hypothesized that miR-506-3p aggravated DBP-induced rat testicular oxidative stress injury via ANXA5/Nrf2/HO-1 signaling pathway. In this study, we found that miR-506-3p significantly downregulated the expression level of ANXA5, then decreased the expression levels of Nrf2 and its downstream target genes, so thereby miR-506-3p might be a damage factor in DBP-induced testicular oxidative damage. In addition, overexpressed ANXA5 weakened the DBP-induced ROS, strengthened the activity of antioxidative stress indexes, and reduced MDA level, which revealed that overexpression of ANXA5 reversed the testicular oxidative stress promoting injury of miR-506-3p in DBP-treated rats via Nrf2/HO-1 signaling pathway.

Since previous studies have illustrated that DBP could induce oxidative stress *in vivo*, leading to the change of proteins in antioxidant pathway, *in vitro* experiment was performed to verify this finding. We firstly redemonstrated the testicular oxidative stress injury after DBP administration by DHE staining in TM3 cells. The generation of ROS decreased cell viability with the increase of DBP concentration. Strikingly, the optimum cell state and significant protein change were showed in DBP 10 mg/L, while the larger doses of DBP could lead to terrible cell viability. Meanwhile, the levels of ANXA5 and Nrf2 relevant genes were also slightly decreased after the management of DBP. By lentiviral transfection to enhance the expression of miR-506-3p, the reduced level of Nrf2 and its downstream antioxidant HO-1, NQO1, and GST were found when recombinant rat ANXA5 was administrated. Collectively, these findings enrich the understanding of DBP in reproductive toxicity, and more comprehensive studies remain to be conducted in the future.

## 5. Conclusion

Overall, this study illuminated that miR-506-3p could aggravate the DBP-treated testicular oxidative stress *in vivo* and *in vitro* by inhibiting the ANXA5 expression and downregulating Nrf2/HO-1 pathway. This study might provide novel understandings in DBP-induced testicular injury therapy.

## Figures and Tables

**Table 1:** The count, viability, and malformation rates of sperm and serum sex hormone levels in each group.

Group	Sperm count (10^5^/mL)	Viability rate (%)	Malformation rate (%)	T (nmol/L)	FSH (IU/L)	LH (IU/L)
Normal	1381.52 ± 124.27	87.36 ± 14.77	4.53 ± 0.88	11.32 ± 1.43	5.99 ± 1.14	12.74 ± 3.08
DBP (800 mg/kg)	667.93 ± 111.79^∗^	74.43 ± 12.51^∗^	17.74 ± 2.72^∗^	3.88 ± 0.72^∗^	13.12 ± 1.85^∗^	15.75 ± 4.25^∗^

^∗^ *P* < 0.05 vs. normal group; *n* = 8 in each group. T: testosterone; FSH: follicle stimulating hormone; LH: luteinizing hormone.

## Data Availability

The data used to support the findings of this study are available from the corresponding author upon request.
